# Impact of high CO_2_ on the geochemistry of the coralline algae *Lithothamnion glaciale*

**DOI:** 10.1038/srep20572

**Published:** 2016-02-08

**Authors:** F. Ragazzola, L. C. Foster, C. J. Jones, T. B. Scott, J. Fietzke, M. R. Kilburn, D. N. Schmidt

**Affiliations:** 1 School of Earth Sciences, University of Bristol, Wills Memorial Building, Bristol BS8 1RJ, UK; 2 Interface Analysis Centre, School of Physics, Tyndall Avenue, Bristol BS8 1TL, UK; 3 GEOMAR | Helmholtz Centre for Ocean Research Kiel, Wischhofstrße 1-3, 24148 Kiel, Germany; 4 Centre for Microscopy, Characterisation and Analysis, University of Western Australia, Australia

## Abstract

Coralline algae are a significant component of the benthic ecosystem. Their ability to withstand physical stresses in high energy environments relies on their skeletal structure which is composed of high Mg-calcite. High Mg-calcite is, however, the most soluble form of calcium carbonate and therefore potentially vulnerable to the change in carbonate chemistry resulting from the absorption of anthropogenic CO_2_ by the ocean. We examine the geochemistry of the cold water coralline alga *Lithothamnion glaciale* grown under predicted future (year 2050) high pCO_2_ (589 μatm) using Electron microprobe and NanoSIMS analysis. In the natural and control material, higher Mg calcite forms clear concentric bands around the algal cells. As expected, summer growth has a higher Mg content compared to the winter growth. In contrast, under elevated CO_2_ no banding of Mg is recognisable and overall Mg concentrations are lower. This reduction in Mg in the carbonate undermines the accuracy of the Mg/Ca ratio as proxy for past temperatures in time intervals with significantly different carbonate chemistry. Fundamentally, the loss of Mg in the calcite may reduce elasticity thereby changing the structural properties, which may affect the ability of *L. glaciale* to efficiently function as a habitat former in the future ocean.

Coralline algae are a major contributor to the marine ecosystems within the photic zone from the cold high latitudes to the tropics^1^,^2^. In cold water environments, they account for 45–56 wt.% of carbonate secreting organisms^2^. Due to their complex 3D structure, they provide important ecosystem goods-and-services by creating key habitats and by producing maerl beds which are economically exploited^3^,^4^,^5^.

Coralline algae are composed of high-Mg calcite with calcification occurring in the cell wall guided by a polysaccharide matrix^6^,^7^. The cell wall structure is well known with an outer zone made of thin needle-shaped crystals tangential to the cell and an inner zone comprised by radial crystals perpendicular to the cell wall^8^. Distinct banding patterns between winter and summer, similar to tree rings, result from changes in cell size and the composition of the calcite^1^. Within each annual band smaller, more calcified, cells are deposited in winter and larger, less calcified, cells are deposited in summer thus highlighting the effect of seasonal environmental change on growth and architecture of the algae. Winter bands are characterized by low Mg concentrations while summer cells have much higher Mg concentrations^9^. The higher Mg during summer and lower concentration during winter are driven by changes in water temperature, based on the endothermic substitution of Mg in Calcite, favouring the Mg substitution at higher temperature. This process has been used to reconstruct past temperatures based on Mg in coralline algae^10^. The uptake of anthropogenic CO_2_ by the ocean has resulted in a significant change in ocean carbonate chemistry^11^,^12^ which has been shown in laboratory cultures to present a challenge to coralline algae^13^. Specifically, the change in CO_2_ has been shown to negatively affect algal growth, calcification^14^, and to alter skeletal chemistry and structure^15^,^16^,^17^,^18^. The future of coralline algae as a habitat former is coupled to the ability of its physical structure to withstand changes within the environment. Changes in the growth structure and the mineralogy will affect this performance. Mg alters the material properties of calcite and hence has important implications for the structural properties of organisms^19^. In sea urchins the high Mg-Calcite phase has higher mean elastic modulus (E) and hardness (H) values compared to pure calcite^20^. Finite Element Modelling of the growth structure of coralline algae specimens cultured under high CO_2_ indicates a reduction of the algae’s ability to withstand predation and erosion due to wave action in response to the changes in growth geometry of the organisms^15^,^21^. However, the added effect of changes in elemental concentration and mineral growth have not been quantified to date and might indicate synergistic or antagonistic impacts of ocean acidification on the structural performance of coralline algae.

Here we assess changes in the geochemistry and crystallography of the non-geniculated cold water coralline alga *Lithothamnion glaciale*, one of the three main maerl bed-forming species in the northern latitudes, grown under natural conditions (summer and winter), in the laboratory under controlled ambient (the control) and at high CO_2_ conditions (589 μatm)^15^. We use Electron Microprobe and nanoscale secondary ion mass spectrometry (NanoSIMS) analyses to generate elemental maps of Mg and Sr at very high spatial resolution on both natural and laboratory-cultured specimens exposed to high CO_2_. These data allow us to evaluate if changes in chemistry might provide an acclamatory response for these species to maintain their structural integrity.

## Results

Bulk XRD confirms that the natural sample is composed of high-Mg calcite (Fig. S1) and that dolomite, as reported in other coralline algae^22^,^23^, was not present in our specimens at the beginning of the experiment. Assessing the presence of dolomite is important as dolomite is 6–10 times less soluble than high Mg-Calcite^22^ and hence is likely to react differently to ocean acidification.

**Figure 1:**
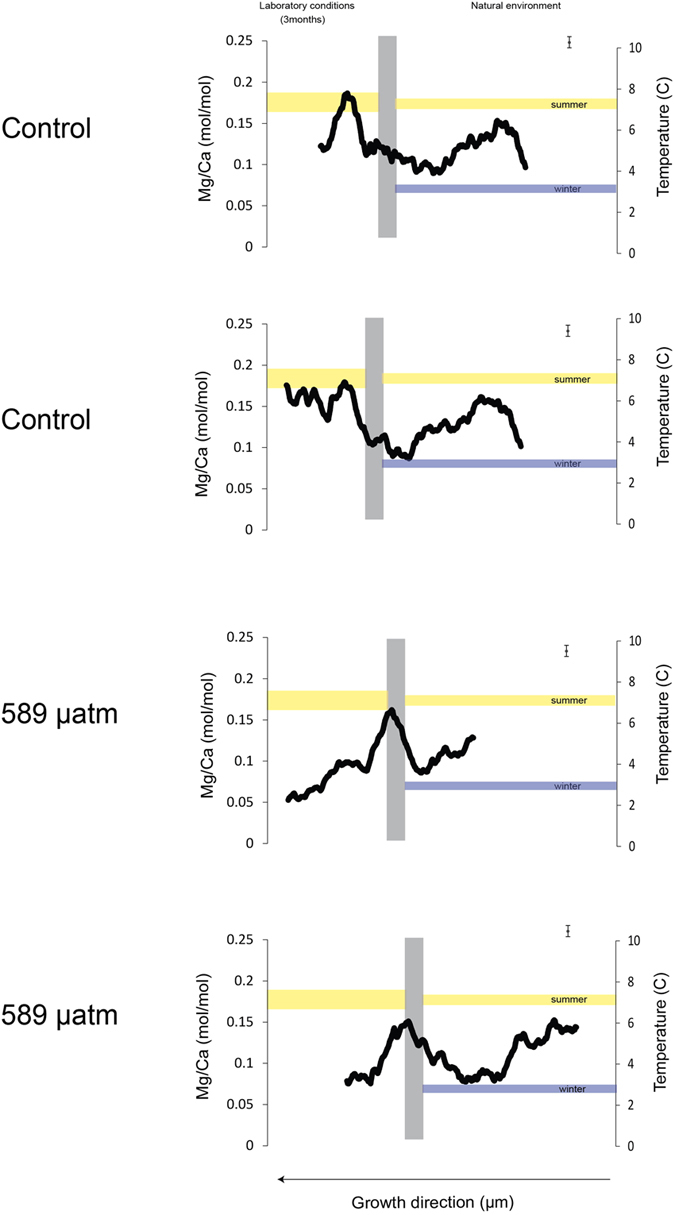
Two Mg/Ca (mmol/mol) transects for each sample were measured using EMPA. The data is represented as a 30 point moving average. Error bars in the top right corner of each graph indicate the uncertainty based on the counting statistics. The grey line demarks the Alizarin staining at the beginning of the experiment. Data left of the line represents growth under laboratory conditions while data right of the graph shows growth in the natural environment. Reconstructed temperature (°C) is shown on the secondary vertical axis using *L. glaciale* temperature equation from Kamenos *et al*. 2008. The yellow horizontal band indicates the culture temperature of the aquaria (7 ± 0.5 °C), the thin lines indicate the seasonal temperate range at the collection site.

Electron microprobe transects document the expected cyclicity in Mg concentration in response to its temperature-dependent Mg incorporation^10^,^24^,^25^ of the pre-cultured material reflecting summer and winter growth (Fig. 1). The Mg/Ca ratio in the carbonate deposited under natural conditions (i.e., prior to the experiment) reflects (0.07 mol/mol to 0.158 mol/mol) the natural Mg variability within the *L. glaciale* thallus and suggest growth temperatures between 2.8 and 6.9 °C, which closely matches natural temperate variability at the site which range between 3.5 to 7.0 °C. It is important to note that not always the same amount of time, i.e. winter and summer growth, was sampled in each individual due to the natural growth variability in each specimen.

The average Mg concentration of the carbonate deposited under experimental conditions was ~0.07 mol/mol lower in the sample cultured under acidified condition (0.08 ± 0.03 mol/mol) (Kruskal-Wallis, df = 3, *p* = 0.001) relative to the samples cultured in control/ambient condition (0.157 ± 0.02 mol/mol). Both control and high CO_2_ conditions samples were grown at the same constant temperature (7 ± 0.5 °C) hence the Mg concentration of the carbonate should be the same. The measured Mg/Ca ratio of the acidified sample would result in a reconstructed temperature of ~ 3.31 ± 1.29 °C while the reconstructed temperature of the control was ~ 6.85 ± 0.91 °C. This discrepancy is far larger than any variability between specimens and hence must have other causes than natural variability.

**Figure 2:**
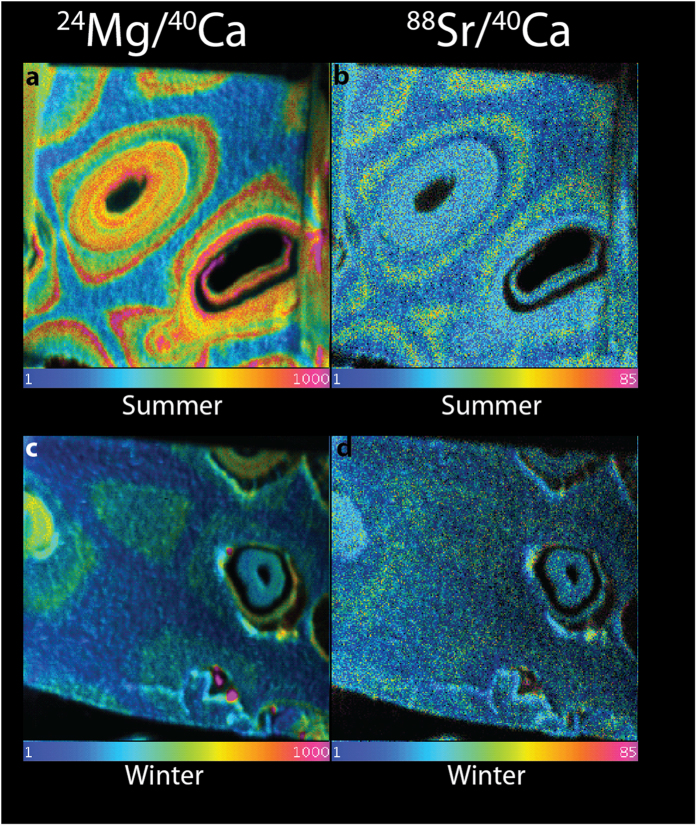
NanoSIMS ratio images of Mg/Ca (left) and Sr/Ca (right) of natural growth in the summer (**a,b**) and winter (**c,d**). The field of view is 20 × 20 um. Note the pronounced banding in both Mg and Sr in the summer samples (top) versus the lower concentrations and less pronounced banding in the winter sample (bottom). The colour scheme represents the ratio of Mg and Sr to Ca, respectively. Blue represents a ratio of 0.0001 Mg/Ca and magenta a ratio of 0.1 for Mg, and 0.0001 and 0.0085 for Sr.

To better understand the reason behind this variability in Mg, we conducted NanoSIMS analyses of the same samples. NanoSIMS increases the spatial resolution of the analysis and creates visual map which can be better linked to the growth of the organism^26^. NanoSIMS analyses of calcite grown in the field during the summer reveal high Mg concentration rings in the cell wall (Fig. 2a), with an average Mg/Ca ratio of the high Mg ring of 0.09 ± 0.004 mol/mol (Table S1). By contrast, the interstitial zone between the cells is largely homogeneous and has a Mg/Ca ratio of ~0.032 ± 0.002 mol/mol. The calcite bands grown during the winter show similar patterns of Mg distribution, but, as expected based on the impact of temperature on Mg incorporation, the concentrations within the wall are lower than those precipitated during the summer; Mg/Ca ratio of 0.04 ± 0.005 mol/mol for the highest concentrations compared to an interstitial ratio of ~0.025 ± 0.0025 mol/mol; (Fig. 2c).

**Figure 3:**
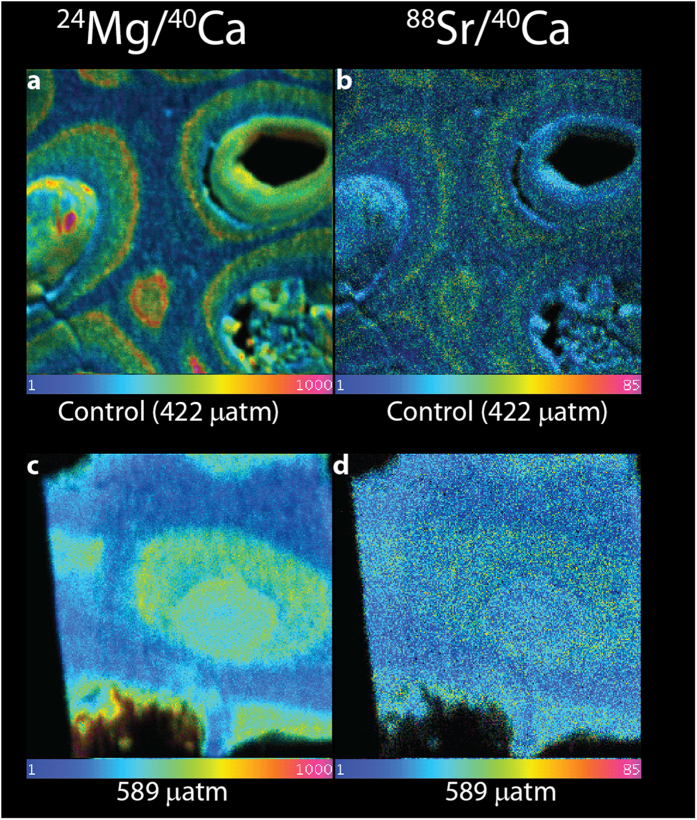
NanoSIMS ratio images of Mg/Ca (right) and Sr/Ca (left) ratios of material deposited during the culturing experiment under (**a,b**) control CO_2_ (422 μatm) conditions (top) and (**c,d**) acidified conditions (589 μatm, bottom). Note the similarity of the banding in the control experiment and natural summer growth (Fig. 4 top) and the distinct loss of banding and overall lower concentrations in the material grown under acidified conditions. The field of view is 20 × 20 um. The colour scale is the same as Fig. 3.

The carbonate precipitated in the lab-based control experiment shows a similar pattern, though lower concentrations, compared to the field summer sample; highest Mg/Ca ratios are 0.057 ± 0.01 mol/mol compared to interstitial values of 0.030 ± 0.0009 mol/mol (Fig. 3a,b). However, the specimen grown under high CO_2_ conditions exhibited significantly lower Mg concentration, specifically in the typical high Mg regions of the control specimen, resulting in a loss of the clear banding for Mg: highest average Mg/Ca is 0.049 ± 0.0054 mol/mol compared to interstitial values of 0.027 ± 0.0009 mol/mol (Fig. 3c). While the concentrations are generally lower for Sr (Fig. 3d), and hence the pattern is less clear, the data suggests a similar trend (Table S1 Fig. S1).

Sr concentrations follow the same pattern as Mg, though the contrast between the layers and between seasons is not as pronounced: Summer and winter samples have highest Sr/Ca of 0.004 ± 0.005 mol/mol and 0.0028 ± 0.002 mol/mol respectively compared to interstitial values of ~0.002 (Fig. 3b,d).

**Figure 4:**
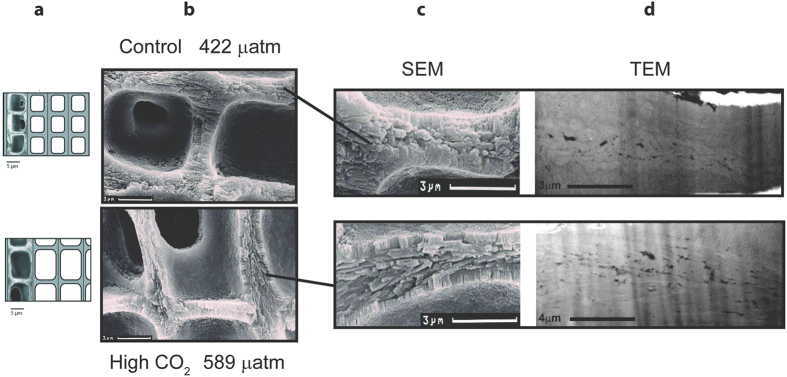
Structural comparison of *L. glaciale* cell wall grown under natural conditions (top) and cultured under high CO_2_ conditions (bottom) using secondary electron microscopy (SEM, middle) (**b,c**) and transmitted electron microscopy (**d**) (TEM, right). The scale model (**a**) of *L. glaciale* on the far left is a modification of Ragazzola *et al*. (2012). Note the TEM images are not at the same scale. The higher CO_2_ growth results in thinner walls and larger cells due to the CO_s_ fertilisation of the photosynthesis (**a,b**). The control sample exhibits a narrow central channel structure, with low porosity and small crystallites with little alignment. In contrast the acidified sample shows strongly oriented, elongate crystals filling the central interstitial zone approximately parallel to the wall surface.

In an attempt to better link the chemical changes to the cell wall growth, structural changes between the algae grown at high CO_2_ and under natural conditions were observed using scanning electron microscopy (SEM) and scanning transmission electron microscopy (STEM) (Fig. 4). Under high CO_2_ conditions algae have notably thinner walls (intra cell walls: control = 0.862 ± 0.08 μm, high CO_2_ = 0.685 ± 0.02 μm; inter cell walls: control = 1.340 ± 0.04 μm, high CO_2_ = 0.829 ± 0.03 μm, Ragazzola *et al*.^15^ and a more angular cell margin (Fig. 4b,c). Closer examination of the wall structure reveals a marked structural difference in the central interstitial zone (reminiscent of the middle lamella). This is most obviously depicted in the TEM (Fig. 4d) which show elongate crystals filling the central interstitial zone of the high CO_2_ structure running strongly oriented, approximately parallel to the wall surface. In contrast, the control sample exhibits a narrower central channel structure, with lower porosity and smaller, less elongate crystallites with a more random orientation.

## Discussion

Over the past decade coralline algae have increasingly been used as archives of palaeoclimate information due to their common occurrence, their longevity and clear incremental growth pattern resulting in easily identifiable annual bands^27^. Mg/Ca ratios have been shown to faithfully record temperature variations in a range of marine calcifiers, such as foraminifers^28^ and coralline algae^27^ as the substitution of Mg in calcite is enhanced at high temperatures.

Our results show heterogeneity of Mg concentrations, both within and between samples, in agreement with other studies^10^,^29^. Specifically, transects across sectioned specimens show differences in the spatial distribution of Mg related to the growth of the algae. The heterogeneity between parts of the thallus, i.e. the none-perfect alignment of the summer winter banding between the specimens, are the result of the three dimensional bio-mineralised nature of the structure which is not symmetrical in every part of the thallus combined with individual growth differences. This results in a variable length of summer (high Mg) and winter (low Mg) bands.

We found that ocean acidification impacts the Mg content of the carbonate significantly more than inter-and intra-specific variations. The Mg content in the carbonate that was deposited under acidified conditions is significantly lower compared to the carbonate deposited under natural conditions (Fig. 1, SI). These results agree with previous studies where Mg content in different species of coralline algae and bryozoans has been shown to decrease in response to acidification^16^,^17^,^30^. In bryozoans, MgCO_3_ decreased by 17% between the control and the acidified condition^30^. Since the samples were kept at constant temperature (7 ± 0.5 °C) during culturing, the lower Mg cannot be related to temperature. This resulting underestimation of temperature of ~ 3.1 °C between carbonate grown under control and acidified conditions undermines the accuracy and viability of using Mg/Ca ratios for temperature reconstructions. While our experiment was run for three months and hence part of this reaction can be the stress of the experiment without time for proper acclimation, our findings raise concerns for the analysis of deep time samples, which lived under significantly higher atmospheric and oceanic CO_2_ conditions than those in our experiments. Our finding of the impact of carbonate chemistry on temperature reconstructions is corroborated by similar effects in both benthic^31^ and planktic foraminifers^32^ with fundamentally different calcification mechanisms compared to coralline algae^33^ in natural environments suggesting that this phenomenon might be of broad scale consequence in many proxy carriers , independent of the biomineralisation mechanism and instead driven by inorganic processes.

At finer spatial scale, the NanoSIMS data shows clear bands of Mg and Sr concentration (Fig. 2), which are lost in the high CO_2_ sample (Fig. 3). Natural summer high Mg bands are more visible than the high Mg bands from the Laboratory control. The experiment was set at average summer temperatures for the location; therefore the natural field growth will have had temperatures which are higher than the seasonal average which would have resulted in higher Mg than the control in small areas which could have influenced the NanoSIMS values. Ideally a future experiment should mimic the natural variability in both temperature and CO_2_ of the natural environment, as FOCE experiments are able to do^34^. Banding has been seen in other organisms for example planktic foraminifera^35^. Here, the formation of the bands has been linked to diurnal changes in calcification chemistry arising from the interplay of algal symbiont photosynthetic activity and net respiration of symbionts and host^36^. This same pattern though can also be seen in non-symbiont bearing species likely reflecting different Mg concentrations of the different growth layers of foraminifera^37^. While photosynthesis could also play a role in the formation of the high Mg bands in coralline algae, it is not a likely explanation for the difference between the control and the acidified sample as previous studies on coralline algae suggest that photosynthesis was not strongly impacted by an increase in CO_2_^14^,^38^,^39^,^40^. Excluding the possible role of photosynthesis, the loss of the Mg bands can be interpreted in two fundamentally different ways: either a biologically controlled loss of Mg in order to decrease the thallus solubility in low saturation waters or a loss of biological control on the biomineralization process. Like all calcifiers, coralline algae strongly discriminate against Mg in seawater as the concentrations in the calcite are several orders of magnitude below seawater values^41^. The role of Mg during biomineralization is still unclear but high Mg concentrations increase calcite solubility^42^,^43^.

In coralline algae, local decalcification occurs when conceptacles (reproductive features) are formed^44^ and hence high Mg would facilitate the dissolution/decalcification of the thallus and hence possibly cell division. Cell division in coralline algae is localized to one or more meristematic regions, regions of undifferentiated cells with light calcification^8^. Calcification involves two successive processes: thin needles appear tangentially in the outer part of the wall and then crystallization develops radially in the cell-frame itself in contact with the plasmalemma^8^. Samples grown under high CO_2_ conditions show these thin needles arranged tangentially to the cells (Fig. 4) without the presence of plates (needles change progressively into plates), thereby resembling the structure of the “younger” cells^8^. Age differences in the SEM and STEM sections can be excluded since the analysed sections were taken from the same distance from the tip. One might speculate that in order to maintain or increase calcification rates at high CO_2_^16^,^45^, the deposition of carbonate in the cell wall is somehow less well controlled hindering development from needles to plates resembling earlier developmental states.

Previous experiments on planktic foraminifera show that Sr incorporation into carbonate, in contrast to Mg, is not primarily controlled by the environment of calcification^46^, but that the uptake is most likely linked to calcification rates^43^,^46^. An important consideration for Sr incorporation is whether Mg concentrations influence Sr uptake. Specifically, the incorporation of the smaller Mg^2+^compared to the Ca^2+^cations in the calcite lattice may distort the crystal lattice which facilitates uptake of larger ions such as Sr^2+^. The incorporation of Sr^2+^could therefore relieve some of the stress caused by the incorporation of Mg^2+^ultimately making the high Mg calcite more stable^42^,^47^. In reverse, the loss of Mg could therefore directly reduce the uptake of Sr and hence result in the loss of the bands as a direct consequence of the decrease in Mg.

It is important to note that mechanical properties in biogenic calcite are strongly related to its Mg content; specifically hardness increases with increasing Mg content^20^,^48^. Elastic modulus and hardness value in non-metals are not truly independent and the two material properties increase together with increase in Mg content in biogenic calcite^49^,^50^. As a consequence of the highly heterogeneous structure of coralline algae, mechanical anisotropy would make the structure highly susceptible to fracture in different directions. The development of high Mg calcite may allow the algae to improve its strength and elasticity in a wave-dominated environment with significant flow^51^. Together with occluded proteins, which reduce the brittleness of the calcite^49^, changes in Mg allow the structure to deform in a plastic manner making it more resistent. In particular in the summer bands, high Mg content would counteract potential weakness due to its thinner cell wall relative to the winter cell.

Previous studies^15^,^16^ have shown an acclimation and resource allocation of *L. glaciale* in long term experiments compared to the short term experiments indicated by a reverse of the cell wall thickness to control conditions but a reduction in growth rate. Importantly though, the lower Mg concentration at the high CO_2_ persist in the long term culture. Even in the long term experiment, we cannot predict if these changes in the elemental composition will be permanent and if so, the extent of how these changes in the elemental composition will affect the coralline algae. The loss in elasticity and hardness (embrittlement) together with the highly heterogeneous structure will potentially increase the susceptibility of the thallus to fracturing in an increasingly wave dominated environment^21^. Increased likelihood of fracturing or damage to the algae will have detrimental impacts to the future ability of coralline algae to perform its ecosystem function. While the organism continues to grow under high CO_2_ conditions its ability to act as a robust structural framework providing a habitat for other marine species might be affected.

## Methods

Specimens of *Lithothamnion glaciale* Kjellman were collected in Kattegat (57° 0.84′ N, 11° 35.10′ E and 57° 0.38′ N, 11° 34.88′ E, i.e. within the same maerl bed but a few kilometres apart) at 20 meters water depth in June 2010 (see Ragazzola *et al*.^15^ for details of culturing methods). The environmental conditions, i.e. water parameters, light etc., were the same in the two locations. The natural temperature variability above the bed ranges from 3.5 to 7.0 °C (see Fig. 1). In order to measure the growth rates (linear extension) under controlled conditions, the laboratory specimens were stained using 0.5 gL^−1^ Alizarin Red staining for 24 hours at 8 °C in 12:12 hours light-dark cycle^52^. From this population of coralline algae, specimens were randomly selected (healthy specimens with no sign of damage or bleaching) and cultured at constant current ocean pCO_2_ conditions (422 μatm) as a control, and at 589 μatm to mimic those expected within the next 50 years^11^,^53^. Each specimen was randomly assigned to an aquarium of the two above treatments. The temperature was also kept constant at 7 ± 0.5 °C, with 20 μmol photons m^−2^ sec ^−1^ in 12 hours light/ dark cycle (using a 39 W fluorescent tubes above each aquarium) to provide irradiance and temperature corresponding to that found at 20 m water depth at Kattegat during the summer season in both aquariums.

For Scanning Electron Microscopy (SEM), samples were examined with a CamScan-CS-44 at Geomar (Kiel). Longitudinal sections of the thalli were cleaned with buffered pH distilled water and dried at 50 °C for 24 hours. The samples were then mounted on aluminium stubs and coated with a gold/palladium mixture (80%/20%).

Samples were prepared for Transmission Electron Microscopy (TEM) by cutting and extracting a thin lamellae of material from selected areas of the specimens using a Helios X600 Dualbeam, a combined electron microscope and focused ion milling instrument fitted with kleindiek nano-manipulator. The lamellae were each 80–100 nm in thickness and the resulting images allow a high resolution assessment of differences in texture and porosity between the samples.

X-ray diffraction (XRD) analysis was carried out at the University of St. Andrews using an automated Philips PW1050 X-ray diffractometer with Philips WinXRD V2 software. Quantification was performed using Rietveld analyses using SiroquantTM software (Sietronics Pty Ltd.)

**Figure 5:**
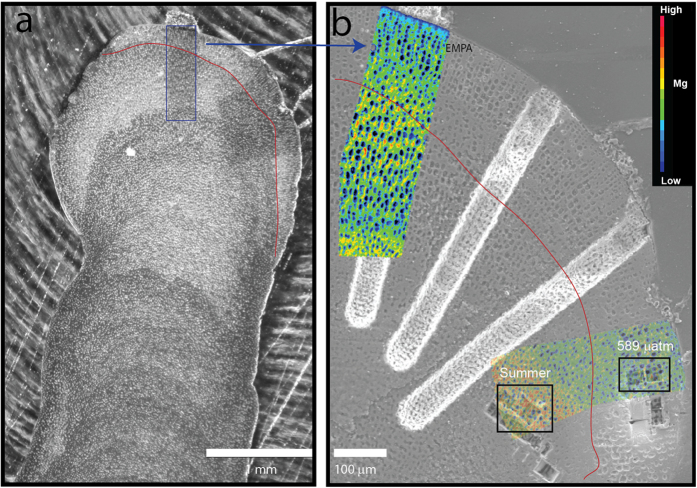
(**a**) SEM image of polished cross section of *L. glaciale* showing material grown under natural conditions (below the red line) and cultured under high CO_2_ conditions (589 μatm, above the red line). Red line indicates the Alizarin staining. Darker areas represent smaller cells and winter growth while lighter areas represent the larger cells grown during the summer. (**b**) Location of the Electron microprobe analysis (EMPA, in colour) and lift outs for TEM and NanoSIMS (summer, natural environment and 596 μatm CO_2_, laboratory experiments). The sample shows clear seasonal growth with lower Mg concentrations (blue colours) in wider bands and higher Mg concentrations (yellow and red colours) in the summer than in the winter.

Elemental distribution was mapped with a JEOL JXA 8200 electron microprobe at GEOMAR, using an accelerator voltage of 15.0 kV, a beam current of 50 nA, beam diameter 2 μm, dwell time 20 msec, and 10 accumulations. Two parallel transects were selected on these maps and the Mg and Ca concentration counts for each transect were extracted (software developed by JEOL, XM-27330 (V01.41) along the line of growth (Fig. 5). Elemental concentrations were calibrated against known standards for Mg (glass VG2 Juan de Fuga = NMNH 111240 -standard reference value: 4.05 wt%) and Ca (Calcite = NMNH 136321-standard reference value: 40.11 wt%). Mg/Ca was converted to temperature using the equation of Kamenos *et al*. 2008 (T°C = 49.40*Mg/Ca −0.92) for *L. glaciale*. Very low Mg concentrations are likely to be influenced by the nature of the sample i.e. high porosity due to high cell density which impacts measurements near the pores as this alters the beam geometry. To minimise the impact of these outliers, we averaged the data using a 30-point running average (Fig. 1).

NanoSIMS ion mapping was performed using the Cameca NanoSIMS 50 at the University of Western Australia. Two samples were taken from material deposited prior to culturing, with one section from the winter, and one from the summer growth indicated by cell habit and elemental distribution in the EMPA scans. Two further samples were extracted from the cultured material; one from the control at 422 μatm and one from the sample exposed to 589 μatm pCO_2_. This enabled us to compare natural material grown during different seasons, and laboratory material grown under different CO_2_ conditions (but the same temperature).

Sections measuring approximately 20 × 20 × 0.2 μm were excavated and lifted out (Fig. 5) of the bulk samples for NanoSIMS analysis. A Pt strap was used to protect the sample and prevent charging. The sections were Pt-welded to a standard 3 mm Cu TEM Omniprobe half disc using a Dualbeam instrument, loaded into a NanoSIMS TEM grid holder, and coated with 10 nm Au to maintain conductivity. Secondary ion images of ^24^Mg, ^44^Ca and ^88^Sr were simultaneously acquired by rastering an O^−^ primary ion beam across the sample surface. High-resolution images from a 20 × 20 μm field of view were acquired using a 1.3 pA beam (with a nominal probe diameter of approximately 300 nm), at a pixel resolution of 256 × 256, with a count time of 100 ms/pixel. Images were processed using the OpenMIMS plugin for ImageJ, utilising the Hue-Saturation-Intensity scale to display the ratio on a colour scale. Ratios were extracted from images by drawing regions of interest on the images, and calculating the ratios from the deadtime-corrected counts for the respective ion species.

## Additional Information

**How to cite this article**: Ragazzola, F. *et al*. Impact of high CO_2_ on the geochemistry of the coralline algae *Lithothamnion glaciale*. *Sci. Rep*. **6**, 20572; doi: 10.1038/srep20572 (2016).

## Supplementary Material

Supplementary Information
