# Supplementary material for: Impact of high CO2 on the geochemistry of the coralline algae Lithothamnion glaciale
Source: Sci Rep. 2016 Feb 8;6:20572. doi: 10.1038/srep20572 (PMC4744931; doi:10.1038/srep20572)
Supplement: Supplementary Information [file srep20572-s1.pdf]

Supplementary Information for

Impact of high CO_2 on the geochemistry of the coralline algae *Lithothamnion glaciale*

Ragazzola, F.[1,5,*], Foster, L.C.[1], Jones, C.J.[2], Scott, T.B[2], Fietzke J[3], Matt R Kilburn[4],

Schmidt, D.N.[1]

[1] School of Earth Sciences, University of Bristol, Wills Memorial Building, Bristol BS8 1RJ, UK
[2] Interface Analysis Centre, School of Physics, Tyndall Avenue, Bristol BS8 1TL, UK
[3]GEOMAR | Helmholtz Centre for Ocean Research Kiel, Wischhofstrße 1-3, 24148 Kiel, Germany
[4]Centre for Microscopy, Characterisation and Analysis, University of Western Australia, Australia
[5]Current address: Institute of Marine Sciences, University of Portsmouth, Ferry Road, Portsmouth PO4 9LY
[*] Corresponding author

Seawater chemistry

Carbonate system

treatment	pH (free scale)	Sal	T (ºC)	TAlk (µmol kg-1)	DIC (µmol kg-1)	HCO_3^- (µmol kg-1)	Ω_{Ca}	Ω_{Ar}	pCO_2 (µatm)
1 (410 µatm)	8.03± 0.05	32.2± 1.2	7.7± 0.2	2311.5± 101.2	2159.45± 86.6	2076.7± 76.6	2.8± 0.33	1.77± 0.2	422± 38.9
2 (560 µatm)	7.90± 0.03	31.3± 0.1	7.6± 0.1	2315.0± 122.7	2216.7± 89.2	2181.8± 106.4	2.08± 0.3	1.31± 0.2	589± 29.6
4 (840 µatm)	7.81± 0.07	31.5± 0.7	7.7± 0.3	2355.1± 96.0	2496.1± 0.1	2226.5± 77.8	1.78± 0.34	1.12± 0.2	755± 118.10
3 (1120 µatm)	7.72± 0.07	31.6± 0.7	7.7± 0.2	2537.7± 97.5	2285.37± 155.9	2403.8± 83.9	1.55± 0.15	0.98± 0.09	1018± 174.8

Carbonate system parameters during the 3 months incubation of *L. glaciale*. All numbers are mean values (n=4) ± STD. The pH, salinity, temperature and total alkalinity (TAlk) were measured while other parameters were calculated (from Ragazzola et al 2012).

Experimental set up

Specimens of *L. glaciale* Kjellman were collected in Kattegat (57° 0.84’ N, 11° 35.10’ E and 57° 0.38’ N, 11° 34.88’ E) at 20 meters depth in June 2010 on board RC Littorina. The selected specimens were randomly assigned in 16, 5L glass aquaria filled up with natural seawater (salinity 32) and bubbled with 4 different CO_2 concentrations (422 µatm, 589 µatm, 755 µatm and 1018 µatm) for 3 months using a CO_2 mixing-facility (KICO2 - Kiel CO_2 manipulation experimental facility, Linde Gas & HTK Hamburg, Germany). The pCO_2 concentration were slowly increased over 1 month, apart from the control, until the desired concentrations were reached. The experimental condition were set at 7 ± 0.5 ºC with 20 µmol photons m^{-2} sec^{-1} in 12 hours light/ dark cycle. For this study only the *L.glaciale* cultured at 589 µatm and the control were used (from Ragazzola et al. 2012).

NanoSIMS elemental ratio

individual ROI results

sample	ROI		Mg/Ca [mol/mol]	2SE [mol/mol]	2SEM [%]	Sr/Ca [mol/mol]	2SE [mol/mol]	2SEM [%]
**Hi CO2**	1	Interstitial	0.0262	0.0002	0.6	0.00237	0.00003	1.3
Image 17	2	Interstitial	0.0279	0.0002	0.7	0.00223	0.00004	2.0
	3	Interstitial	0.0274	0.0002	0.8	0.00228	0.00005	2.3
	4	HighMg	0.0446	0.0004	1.0	0.00298	0.00007	2.2
	5	HighMg	0.0513	0.0002	0.4	0.00307	0.00003	1.1
	6	HighMg	0.0558	0.0011	2.0	0.00320	0.00010	3.1
	7	HighMg	0.0449	0.0007	1.5	0.00321	0.00015	4.5
		highest	0.0614			0.00411		
**Summer**	1	Interstitial	0.0305	0.0003	0.9	0.00223	0.00004	2.0
Image 28	2	Interstitial	0.0339	0.0003	1.0	0.00231	0.00007	3.0
	3	Interstitial	0.0330	0.0009	2.6	0.00232	0.00013	5.5
	4	HighMg	0.0912	0.0015	1.6	0.00409	0.00014	3.5
	5	HighMg	0.0887	0.0023	2.6	0.00419	0.00027	6.3
	6	HighMg	0.0891	0.0016	1.8	0.00392	0.00016	4.2
	7	HighMg	0.0971	0.0015	1.6	0.00428	0.00016	3.7
		highest	0.1227			0.00505		
**Winter**	1	Interstitial	0.0261	0.0002	0.8	0.00253	0.00004	1.6
Image 40	2	Interstitial	0.0226	0.0003	1.2	0.00244	0.00006	2.4
	3	Interstitial	0.0275	0.0006	2.1	0.00256	0.00013	5.1
	4	HighMg	0.0443	0.0004	0.9	0.00292	0.00007	2.3
	5	HighMg	0.0496	0.0010	2.1	0.00297	0.00007	2.3
	6	HighMg	0.0390	0.0009	2.4	0.00258	0.00009	3.4
	7	HighMg	0.0392	0.0006	1.5	0.00258	0.00007	2.7
		highest	0.0552			0.00493		
**Control**	1	Interstitial	0.0304	0.0002	0.6	0.00262	0.00003	1.2
Image 42	2	Interstitial	0.0295	0.0005	1.8	0.00256	0.00012	4.8
	3	Interstitial	0.0314	0.0016	5.1	0.00265	0.00035	13.2
	4	HighMg	0.0555	0.0006	1.1	0.00273	0.00004	1.5
	5	HighMg	0.0509	0.0005	0.9	0.00268	0.00004	1.5
	6	HighMg	0.0517	0.0006	1.2	0.00253	0.00006	2.2
	7	HighMg	0.0732	0.0011	1.6	0.00375	0.00009	2.5
		highest	0.1092			0.00610		

Table SI A: individual ROI results

means of ROI

sample	ROI		Mg/Ca [mol/mol]	SD [mol/mol]	RSD [mol/mol]	Sr/Ca [mol/mol]	SD [mol/mol]	RSD [%]
**Hi CO2**	**mean**	**interstitial**	**0.0272**	**0.0009**	**3.2**	**0.00229**	**0.00007**	**3.0**
Image 17	**mean**	**high**	**0.0492**	**0.0054**	**10.9**	**0.00312**	**0.00011**	**3.4**
**Summer**	**mean**	**interstitial**	**0.0325**	**0.0018**	**5.6**	**0.00229**	**0.00005**	**2.1**
Image 28	**mean**	**high**	**0.0915**	**0.0039**	**4.2**	**0.00412**	**0.00015**	**3.8**
**Winter**	**mean**	**interstitial**	**0.0254**	**0.0025**	**9.7**	**0.00251**	**0.00006**	**2.5**
Image 40	**mean**	**high**	**0.0430**	**0.0050**	**11.7**	**0.00276**	**0.00021**	**7.7**
**Control**	**mean**	**interstitial**	**0.0304**	**0.0009**	**3.1**	**0.00261**	**0.00005**	**1.8**
Image 42	**mean**	**high**	**0.0578**	**0.0105**	**18.1**	**0.00292**	**0.00056**	**19.1**

Table SI B: means of ROI

Table S1: Elemental ratios extracted from regions-of-interest (ROI) within NanoSIMS images. Uncertainties of individual ROI data (part A) are given as 2SE and related to the analytical uncertainty within each ROI. Uncertainties of the means of ROI (part B) are reported as SD representing the variability within the sample.

XRD of *Lithothamion glaciale*

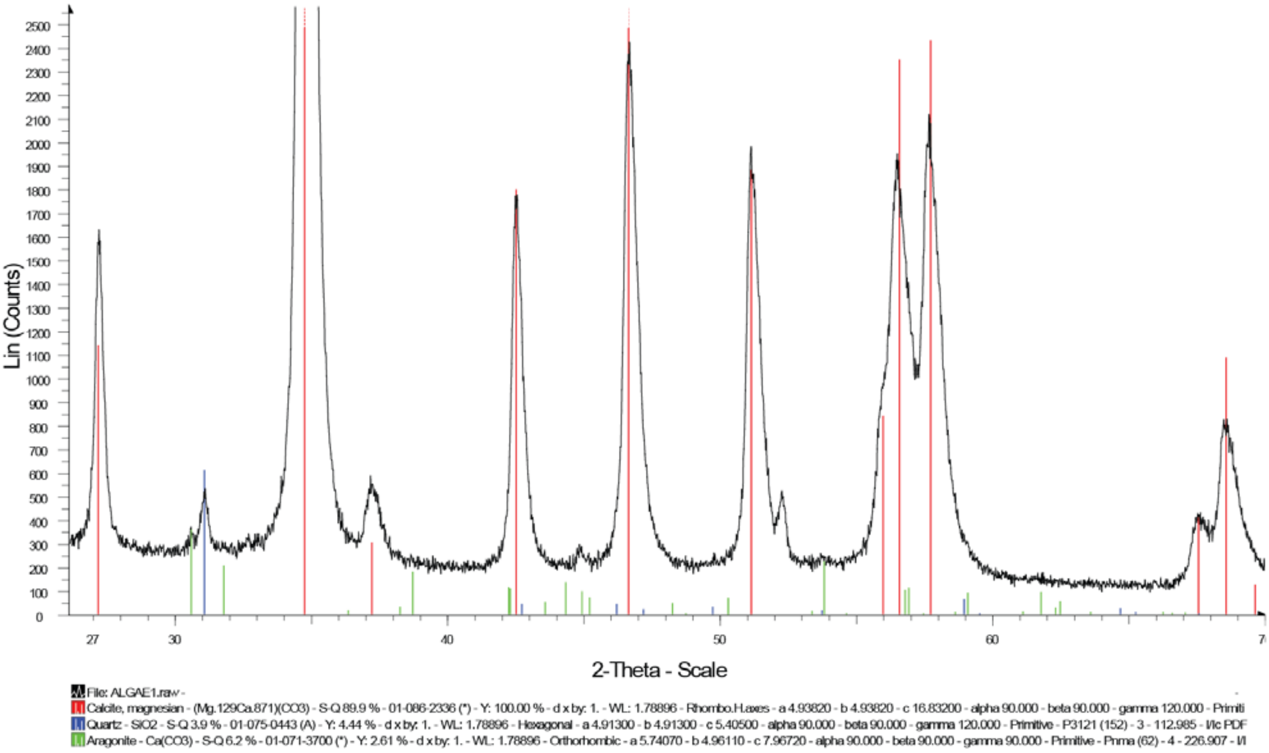

Fig S1: XRD spectrum, showing no sign of dolomite. This is important since in the tropical coralline algae *Porolithon onkodes* the presence of dolomite in the skeleton lowers the dissolution rates by 6-10 times (Nash *et al.*, 2012).

NanoSIMS Mg/Ca and Sr/Ca ratios

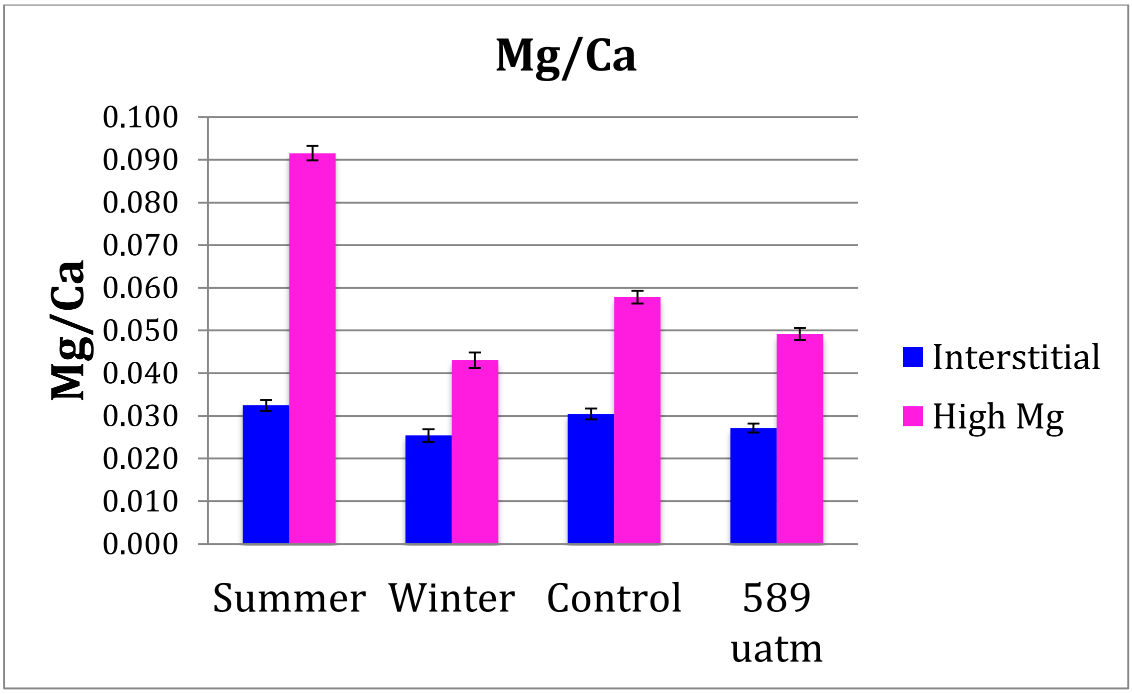

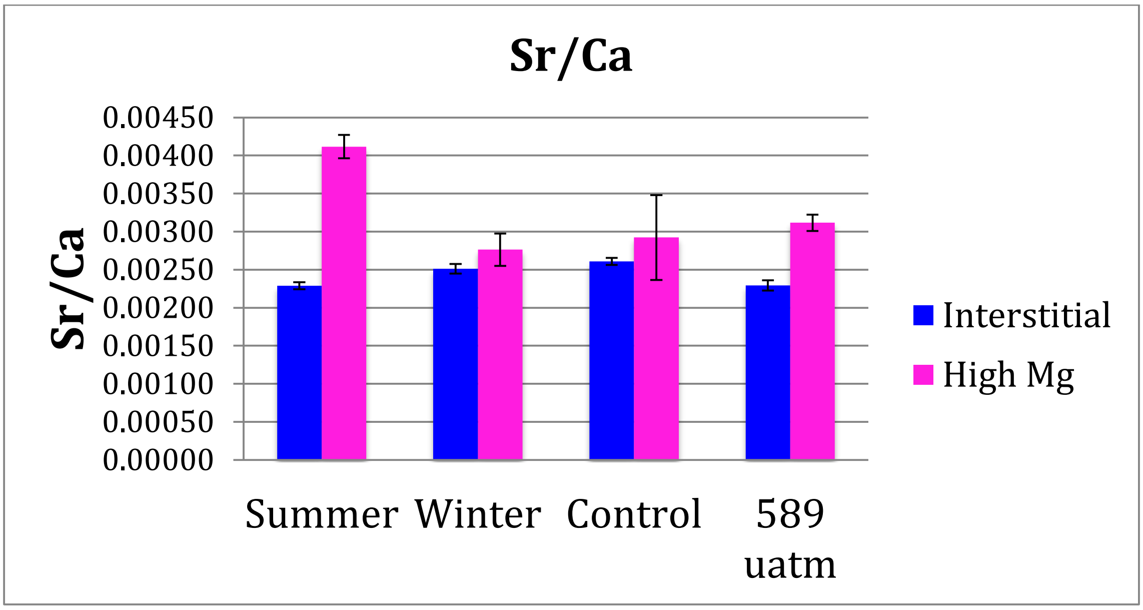

Fig S2: Mg/Ca and Sr/Ca ratios measured by NanoSIMS. The ratios were determined by extracting deadtime-corrected counts from regions-of-interest (ROI) in the ion images, and processing in a spreadsheet. Errors are expressed as the standard deviation

of the pixels within the ROIs. “High Mg” refers to the highest measured ratios within a given image pair.
